# Analysis of cattle olfactory subgenome: the first detail study on the characteristics of the complete olfactory receptor repertoire of a ruminant

**DOI:** 10.1186/1471-2164-14-596

**Published:** 2013-09-02

**Authors:** Kyooyeol Lee, Dinh Truong Nguyen, Minkyeung Choi, Se-Yeoun Cha, Jin-Hoi Kim, Hailu Dadi, Han Geuk Seo, Kunho Seo, Taehoon Chun, Chankyu Park

**Affiliations:** 1 Department of Animal Biotechnology, Konkuk University, 263 Achasan-ro, Gwangjin-gu, Seoul 143-701, Korea; 2 College of Veterinary Medicine, Chonbuk National University, Jeonju, Jeollabuk-do, South Korea; 3 Colleges of Veterinary Medicine, Konkuk University, Seoul, South Korea; 4 College of Life Sciences and Biotechnology, Korea University, Seoul, South Korea

**Keywords:** Olfactory receptor, Cattle, Olfaction, OR genes

## Abstract

**Background:**

Mammalian olfactory receptors (ORs) are encoded by the largest mammalian multigene family. Understanding the OR gene repertoire in the cattle genome could lead to link the effects of genetic differences in these genes to variations in olfaction in cattle.

**Results:**

We report here a whole genome analysis of the olfactory receptor genes of *Bos taurus* using conserved OR gene-specific motifs and known OR protein sequences from diverse species. Our analysis, using the current cattle genome assembly UMD 3.1 covering 99.9% of the cattle genome, shows that the cattle genome contains 1,071 OR-related sequences including 881 functional, 190 pseudo, and 352 partial OR sequences. The OR genes are located in 49 clusters on 26 cattle chromosomes. We classified them into 18 families consisting of 4 Class I and 14 Class II families and these were further grouped into 272 subfamilies. Comparative analyses of the OR genes of cattle, pigs, humans, mice, and dogs showed that 6.0% (n = 53) of functional OR cattle genes were species-specific. We also showed that significant copy number variations are present in the OR repertoire of the cattle from the analysis of 10 selected OR genes.

**Conclusion:**

Our analysis revealed the almost complete OR gene repertoire from an individual cattle genome. Though the number of OR genes were lower than in pigs, the analysis of the genetic system of cattle ORs showed close similarities to that of the pig.

## Background

Mammalian odorant olfactory receptor genes were initially reported in rodents around 2 decades ago [1]. In mammals, odorant molecules are detected by olfactory receptors (ORs), which belong to the G-protein-coupled receptor superfamily and contain 7 transmembrane domains [1]. Olfaction involves the specific binding of volatile odorant molecules to dedicated ORs expressed by olfactory sensory neurons (OSNs) in the olfactory epithelium and the transmission of electrical signals to the olfactory bulb [2-5]. The genes encoding OR proteins comprise the largest superfamily in the mammalian genome. Using available genome sequences, studies have been conducted to elucidate OR subgenomes in diverse species including pigs [6], mice [7], humans [8], dogs and rats [9] and platypus, opossum, macaque and cattle [10], frogs and chickens [11], and fishes [12]. The results showed that there were large variations in the size of OR gene repertoires. However, we felt that further refinement in the accuracy and details on cattle OR genes could significantly improve current understanding on the olfactory system of cattle.

Understanding OR repertoires and individual variations among the same species may be important for determining the potential of individual animals associated with economic traits in livestock animals although such studies have not been reported. Cattle are globally important for the production of animal proteins and may be an attractive animal model to study olfaction and its influences on animal behavior. Characterization and classification of the bovine OR gene repertoire with high accuracy could help to better understand the relationship between animal behavior and olfaction in domestic animals and the characteristics of OR systems in artiodactyl mammals. In addition, a comparison of OR gene repertoires among other animals with diverse physiological characteristics could reveal evolutionary changes in the genetic component of olfaction under different conditions. In this study, we analyzed the cattle genome assembly UMD 3.1, identified the nearly complete olfactory subgenome of cattle, and compared it with other species.

## Methods

### Animals

Tissues from 9 Hanwoo (Korean native cattle) and 9 Black Angus and frozen semen from 4 Holstein animals were purchased from local markets and a breeding company.

### DNA isolation

Animal tissues were incubated with a lysis buffer (10 mM of Tris–HCl pH 8.0, 0.1 M of EDTA) containing 0.5% SDS and 5 μl of 20 mg/ml proteinase K (Promega, USA) at 55°C for 6 hrs. Semen samples were washed with 1X PBS (phosphate buffered saline) and dipped into liquid nitrogen followed by hot water for several times to make the membrane surrounding the acrosome become permeable prior to incubate with the lysis buffer. DNA was isolated from the tissues incubated with the lysis buffer according to a standard protocol [13].

### PCR amplification

PCR reactions using genomic DNA were performed in a 20 μl reaction containing 50 ~ 100 ng DNA, 0.2 μM primers (Additional file 1), 200 μM dNTPs, and 0.5 U LA *Taq* DNA polymerase (Takara, Japan) in a PCR reaction buffer (1.5 mM MgCl_2_). PCR consisted of an initial denaturation step at 94°C for 5 min, followed by 35 cycles of denaturation at 94°C for 30 s, 1 min at specific annealing temperature and specific extension time ~1 min 30 sec at 72°C for each primer pair (Additional file 1) in a Thermocycler 3000 (Biometra, Germany). A final extension step was performed at 72°C for 10 min. Aliquots of PCR products were subjected to electrophoresis in 1% agarose gels in 1 × TAE running buffer for 30 min at 100 V, stained with ethidium bromide (Sigma-Aldrich, USA), and visualized under UV light. The specificity of PCR amplicons was confirmed by analyzing their sequence on an automated DNA Analyzer 3730XL (Applied Biosystem, USA).

### Detection of OR genes in the cattle genome

OR sequences were identified using a method previously used to search for OR genes in several species [6-8]. We retrieved the bovine draft genome sequences (UMD 3.1) from the National Center for Biotechnology Information (NCBI). Next, we perform a translated basic local alignment search tool (TBLASTN) search to identify regions containing OR-related sequences that had at least 2 of the following conserved motifs: MAYDRYVAIC (TMIII), KAFSTCASH (TMVI), and PMLNPFIY (TMVII), or their variants showing a maximum of 50% difference from the conserved motifs. From the identified regions of the BLAST matches, we extended 1 kilobase (kb) both upstream and downstream to predict OR coding sequences. From the analysis, we identified 1,423 OR candidate sequences that were 2 kb in length and translated to amino acid sequences in all 6 frames. We then retrieved 24,809 OR protein sequences from 222 species in NCBI and performed a protein BLAST (BLASTP) analysis against the translated OR candidate sequences to determine the positions of the start and stop codons of the open reading frames (ORFs) considering the structural similarity to known OR proteins. For sequences that deviated from the sequences of reported OR proteins, the methionine and stop codon most similar in sequence context to those of the coding sequences of known OR proteins were selected as the start and end of the coding regions. We again performed a TBLASTN analysis against the 1,423 sequences to evaluate for the presence of all 4 conserved motifs [GN, MAYDRYVAIC (TMIII), KAFSTCASH (TMVI), and PMLNPFIY (TMVII)]. Candidate sequences were considered “functional ORs” if they were at least 300 amino acids long without any interrupting stop codons and/or frameshifts within the ORFs, “OR pseudogenes” if they were at least 300 amino acids in length but contained stop codons or frameshifts within the ORFs, or “partial ORs” if they were shorter than 300 amino acids but matched the sequences of known OR genes. Sequences similar to non-OR G-protein-coupled receptors or partial sequences were removed from our analyses, leaving 1,071 putative OR genes (including pseudogenes).

### Phylogenetic analysis and classification

We retrieved 457; 908; 845; and 1,301 OR sequences from human, mouse, dog, and pig, respectively, and combined them with cattle (1,071 putative OR genes from 1,423 putative genes minus 352 partial genes), then we aligned these 4,582 OR genes together using CLUSTALW [14]. An unrooted phylogenetic tree was constructed after 1,000 rounds of bootstrapping. This tree was used for classifying OR gene families and subfamilies. Cattle OR sequences that did not form a cluster with any reference ORs from the other 4 species were additionally classified using a sequence similarity matrix (data not shown) in which 40% and 60% amino acid similarity were used as the thresholds to distinguish between families and subfamilies, respectively, as previously described [15].

### OR gene nomenclature

For naming cattle OR genes, we followed the OR gene classification system described by Glusman et al. [15]. Functional cattle OR genes were named “bORmXn” whereas pseudogenes were named “bORmXnP”, where “b” stands for *B. taurus*, “OR” is the root name indicating an olfactory receptor, “m” is an integer representing the family that the gene belongs to, “X” is a single letter denoting the subfamily of the gene, and “n” is an integer representing an individual family member. The names of the cattle OR sequences were devised considering their phylogenetic relationships. For example, bOR1A1 is an OR gene of family 1, subfamily A, and is the first member of this subfamily. In the case of pseudogenes, a name such as bOR1G3P indicates an OR pseudogene of family 1, subfamily G, that is the third member of this subfamily. Duplicated genes with the exact same coding sequences were indicated by adding the suffix A or B at the end of their names, i.e., bOR7A17A and bOR7A17B.

### Identification of cattle-specific OR genes

Multispecies OR gene clustering analysis was performed with OR protein sequences from humans, dogs, mice, pigs, and cattle using the OrthoMCL 3 software [16], in order to group them on the basis of sequence similarity and divergence. In total, 751 clusters were formed from 4,582 sequences. The cutoff value for a cluster was 60% similarity at the level of the protein sequence, resulting in sequences with greater than 60% similarity being clustered together regardless of the species of origin.

### Detection of conserved motifs and patterns

To detect conserved motifs in predicted OR protein sequences, sequence logos were generated from an alignment of functional OR gene sequences using the WebLogo program [17]. The PRATT [18] program from the Pattern Discovery Platform was used to define cattle OR-specific patterns with the criteria listed in Additional file 2.

## Results

### Cattle OR gene repertoire and their distribution in the cattle genome

**Figure 1:**
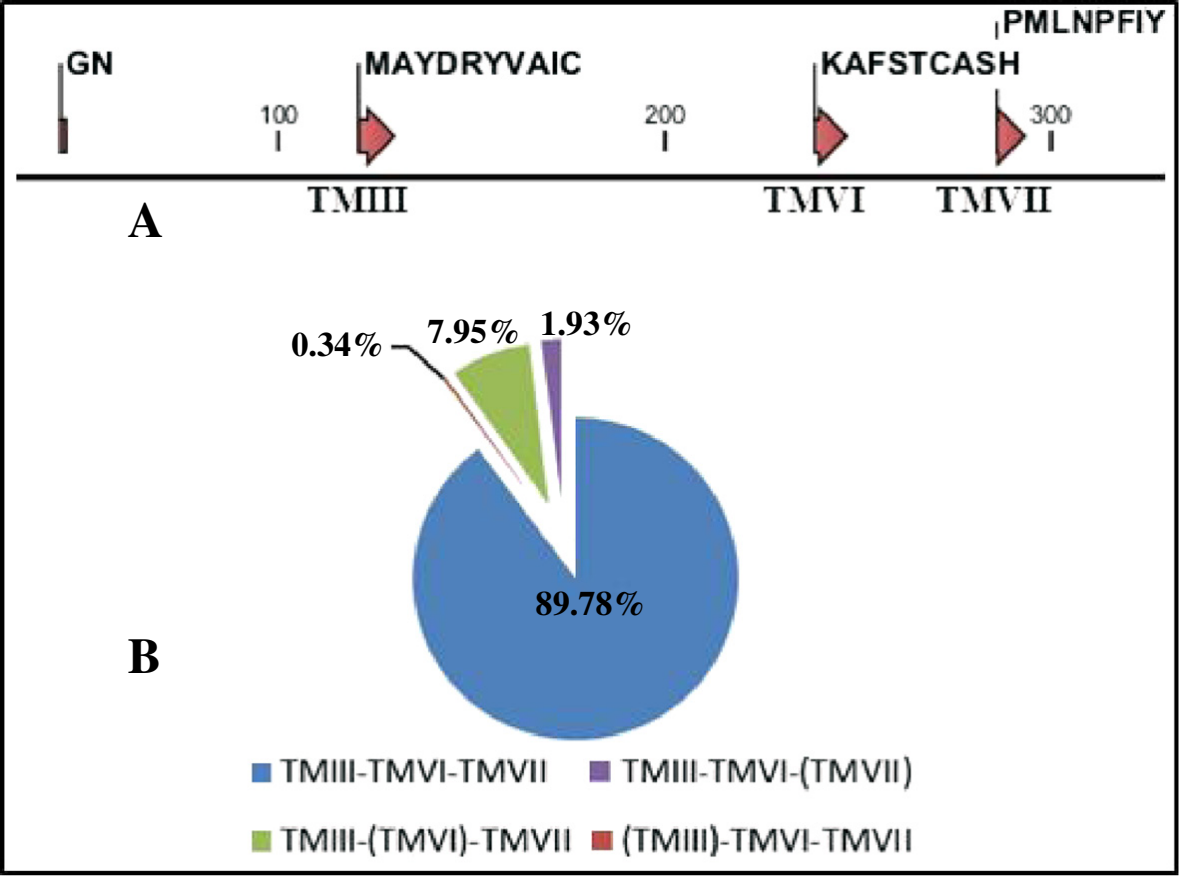
**Conserved OR-specific motifs used to identify OR genes in the cattle genome, and the frequency of sequences with or without these motifs. (A)** Amino acid sequences of the OR-specific motifs are shown. The numbers indicate the positions of amino acids. TM, transmembrane domain. **(B)** Proportional distribution of the 881 functional OR amino acid sequences identified by their OR motif-containing patterns. The motifs within parentheses were absent. GN motifs were observed with or without variations.

Similar to our previous study on the identification of OR genes from the pig genome [6], the 4 conserved motif sequences, GN, MAYDRYVAIC, KAFSTCASH, and PMLNPFIY, which are common to mammalian OR genes, were used to search for the full repertoire of ORs in the cattle genome (Figure 1A). We identified 1,423 OR gene-related sequences with lengths of 900–1,000 base pairs (bp). Among them, 881 OR sequences were identified as functional and 190 were identified as pseudogenes. From 881 OR functional sequences, we obtained 89.78% of the sequences containing all 4 OR motifs and the rest were missing 1 of the conserved motifs (Figure 1B).

**Figure 2:**
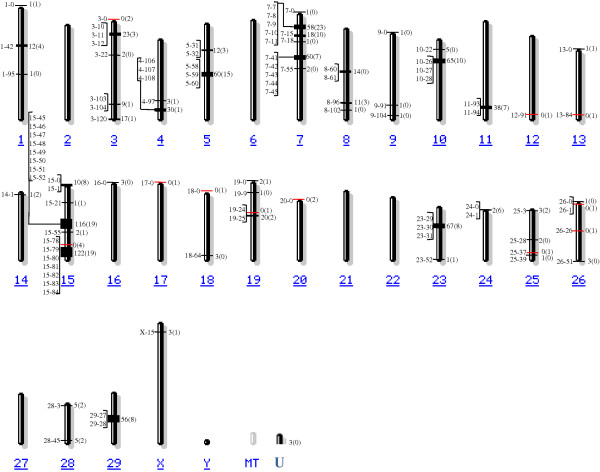
**Chromosomal distribution of cattle OR genes.** Cattle OR genes were mapped to 49 regions across 26 chromosomes. The number of functional and pseudo OR genes at each cluster is indicated to the right of the chromosomes without and with parentheses, respectively. Clusters with and without functional OR genes are indicated by black and red lines, respectively. The position of each cluster is shown to the left of the chromosomes in Mb. Cluster naming scheme A-B: A, chromosome name and B, distance (Mb) from the top of that chromosome. “U” indicates a group of sequences with no chromosome assignment in the cattle genome assembly UMD3.1. Chromosome figures were modified from [19].

The locations of the OR genes were analyzed as per their relative positions in the cattle genome by grouping them into gene clusters according to their positional proximity. If the coding sequences of the OR genes were more than 1 megabase (Mb) apart, they were considered to be present on different clusters. Of the 1,071 functional genes and pseudogenes, 1,068 were mapped to 49 different chromosomal regions across 26 cattle chromosomes and the remaining 3 were located on chromosome U, which contains unmapped contigs lacking any chromosome information (Figure 2). Except for chromosomes 2, 6, 21, 22, 27, and Y, which were devoid of OR genes, all other chromosomes contained 1 to 303 OR genes (Table 1). Chromosome 15 had the largest number of OR functional genes (n = 251), followed by chromosomes 7, 5, 10, and 23. Accordingly, chromosome 15 contained the largest number of OR subfamilies with 100 subfamilies, while only a single subfamily was present on both chromosomes 12 and 17 (Table 1).

**Table 1:** Composition of OR genes for each cattle chromosome

**Chromosome number**	**No. of functional genes**	**No. of pseudogenes (%)**		**Total**	**No. of subfamilies**
1	14	5	(26)	19	5
2	0	0		0	0
3	51	7	(12)	58	18
4	33	2	(6)	35	9
5	72	18	(20)	90	12
6	0	0		0	0
7	140	40	(22)	180	42
8	26	3	(10)	29	5
9	3	0	(0)	3	2
10	70	10	(13)	80	19
11	38	7	(16)	45	12
12	0	1	(100)	1	1
13	1	2	(67)	3	3
14	1	2	(67)	3	3
15	251	52	(17)	303	100
16	3	0	(0)	3	3
17	0	1	(100)	1	1
18	3	1	(25)	4	2
19	23	4	(15)	27	10
20	0	2	(100)	2	2
21	0	0		0	0
22	0	0		0	0
23	68	9	(12)	77	29
24	2	6	(75)	8	6
25	6	3	(33)	9	5
26	4	2	(33)	6	6
27	0	0		0	0
28	10	4	(29)	14	10
29	56	8	(13)	64	10
X	3	1	(25)	4	4
Y	0	0		0	0
U	3	0	(0)	3	3
Total	881	190	(18)	1,071	

Note: In the case of the absence of both OR functional genes and pseudogenes, the pseudogene percentage was not indicated.

The number of OR genes at individual OR gene clusters ranged from 1 to 122 per cluster (Additional file 3). Due to the presence of a large number of OR genes in the genome, the number of pseudogenes was also high (n = 190). More details on the distribution and sequence information of OR functional genes and pseudogenes in the cattle genome is described in Additional file 4.

### Classification of OR gene repertoires

OR genes are the largest gene superfamily in the mammalian genome, containing more than 1,000 genes in certain species [6,7,9], and ORs with more than 60% identity in protein sequence are suggested to recognize odorants with related structures [20,21]. Therefore, studies of OR genes require systematic classification according to their structural or functional similarity. The identified cattle OR genes were classified into families and subfamilies according to the results of phylogenetic analyses and their sequence similarity as described in the Methods. The results showed that the cattle OR repertoire is comprised of 18 families (4 Class I and 14 Class II) and 272 subfamilies, showing that the family diversity of OR molecules in cattle is higher than in mouse but lower than in pigs, dogs, humans, and rats (Additional file 5).

It is interesting to note that humans and dogs have a larger number of OR subfamilies (n = 300) than that of cattle (n = 272), suggesting that the sequence diversity of OR genes in cattle is more limited. However, the diversity of OR genes in humans is due to the degeneration or pseudogenization of OR genes (52% pseudogenes), and thus functional diversity is much lower in humans than in cattle. As cattle and dogs have a similar number of functional and pseudo OR genes (Table 2), our results showed that actual functional diversity of OR genes in cattle is slightly lower than that of dog.

**Table 2:** Differences in the frequencies of functional OR genes among different species

**Species**	**No. of functional genes (%)**	**No. of pseudogenes**
Pig	1,113 (86)	188
Cattle	881 (82)	190
Rat	1,201 (80)	292
Dog	872 (80)	222
Mouse	1,037 (75)	354
Zebrafish	102 (74)	35
Human	388 (48)	414
Frog	410 (46)	478
Pufferfish	44 (45)	54
Chicken	82 (15)	476

Note: Except for cattle, data were from Niimura and Nei [22] and Nguyen et al. [6].

The number of OR genes belonging to each subfamily may represent the importance of specific subfamilies for the species because OR gene subfamilies that are important for the survival of the species are likely to expand in the genome through evolution. Therefore, we counted the number of ORs in each subfamily (Additional file 6). The diversity of single OR gene subfamilies in cattle (n = 107) was significantly lower than in pigs (n = 146). However, the number of OR genes for bOR7A, the largest subfamily in cattle, (n = 63) was larger than in pigs (n = 52), suggesting the specific subfamily expansion in cattle.

While most subfamilies had 1 to 6 members, 5 subfamilies (bOR1O, bOR4R, bOR7A, bOR8G, and bOR9M) had more than 20 genes each. We suspect that this may be similar in pigs and may suggest a common characteristic of OR repertoires in the artiodactyl lineage. We compared the expanded OR subfamilies among cattle, pigs, dogs, and humans to evaluate the sharing of this expansion. The results showed that all 5 expanded subfamilies in cattle also showed family expansion in pigs and dogs but not in human (Additional file 7).

### Distribution of OR subfamilies within the OR gene clusters in cattle

To study the OR gene density across the cattle genome, the chromosomal locations of all OR gene members of the 272 cattle OR subfamilies were analyzed (Table 1). The largest OR gene cluster in the cattle genome was the cluster “23–29” on chromosome 23, which contained 41 OR genes making up 18 subfamilies. We observed that 228 (83.82%) subfamilies were encoded by genes at a single chromosomal cluster (Additional file 3), suggesting possible functional similarities among OR genes within a cluster, which is consistent to analysis results of OR genes in other species such as humans [8], mice [7], and pigs [6].

When we determined the subfamily composition of individual OR gene clusters, the number of subfamilies within a cluster ranged from 1 to 51 (Additional file 3). Approximately 32.65% (16/49) of the OR clusters encoded only 1 OR subfamily, while 67.35% of clusters (33/49) encoded OR genes of more than 2 subfamilies. In terms of the general characteristics of the OR subgenome in cattle including the number of functional OR genes within a cluster, the number of clusters within a subfamily, and the number of subfamilies within a cluster (Additional file 3) were consistent with those reported for other species including pigs, mice, and humans [6-8].

### Analysis of OR gene duplication and copy number variation in the cattle genome

Gene duplication plays an important role in establishing the biological characteristics or diversity of organisms during evolution [23-25]. Identification of gene duplication with the exact sequence identity is likely to be evidence of recent duplication events [23]. We identified 2 such OR genes in the cattle genome (Additional file 8). The gene bOR7A17 was found in 2 locations and was named as bOR7A17[A and B], and bOR1O1 from 2 locations was named bOR1O1[A and B]. The duplication events consisted of 1 intra- and 1 inter-chromosomal duplication (Additional file 8). To eliminate the possibility that the duplications were caused by errors in the genome assembly, we amplified the duplicated OR genes using PCR primers specific for neighboring sequences of duplicated OR genes which have different flanking sequences. We were able to amplify both copies of the OR gene bOR7A17 (Additional file 9), confirming that this duplication is real. However, we were unable to amplify the duplicated copy of bOR1O1, bOR1O1B, by PCR, from our test animals (data not shown), suggesting either the presence of OR gene copy number variations (CNV) in the genomes between the animals used for PCR in this study and for the genome sequencing project or possible errors from the genome assembly.

Gene duplication is one of the major causes of creating gene copy number variations in the genome. To obtain a snapshot on OR gene CNV for cattle, we selected three additional pairs of OR loci with at least 99% identity in nucleotide sequences which indicate recent gene duplication events. Then a total of 10 OR loci (5 pairs) were subjected to locus specific PCR against our animal panel consisting of three breeds, Korean native cattle, Black Angus and Holstein. Three OR loci bOR1O2, bOR1O4 and bOR9M7, showed the presence of either breed or individual specific CNVs (Table 3). For instance, the OR locus bOR9M7 were found in genomes of 4/9, 5/9 and 0/4 in Korean native cattle, Black Angus and Holstein, respectively. Consistent to the breed nature of Holstein cattle which is highly inbred, the animals showed all or none amplification patterns for all three CNV-associated OR loci without individual variations. However, for both Korean native cattle and Black Angus, CNVs were identified among individuals within the breeds.

**Table 3:** Analysis of the copy number variations for 10 cattle OR genes using PCR against 22 individuals from three different breeds

**OR Loci**	Number of samples with specific amplification (%)*		
	**Korean native cattle**	**Black Angus**	**Holstein**
bOR1O1A^a^	8/9 (89)	8/9 (89)	4/4 (100)
bOR1O1B^a^	0/9 (0)	0/9 (0)	0/4 (0)
bOR1O2^b^	9/9 (100)	8/9 (89)	4/4 (100)
bOR1O4^b^	8/9 (89)	7/9 (78)	4/4 (100)
bOR2AK2^c^	9/9 (100)	9/9 (100)	4/4 (100)
bOR2AK3^c^	9/9 (100)	9/9 (100)	4/4 (100)
bOR7A17A^d^	9/9 (100)	9/9 (100)	4/4 (100)
bOR7A17B^d^	9/9 (100)	9/9 (100)	4/4 (100)
bOR9M7^e^	4/9 (44)	5/9 (56)	0/4 (0)
bOR9M8^e^	9/9 (100)	9/9 (100)	4/4 (100)

[*] Number of samples successfully amplified for each locus out of a total number of PCR-subjected samples. The same superscript indicates pairs of ORs with nucleotide sequence identity greater than 99%.

### Patterns of characteristic amino acid motifs in cattle OR proteins

Using the criteria in Additional file 2, we carried out a pattern discovery analysis for cattle OR genes. Table 4 shows 5 motif patterns identified from 4 conserved transmembrane domains of cattle OR genes, TMII, TMIII, TMVI, and TMVII. The motif patterns are similar to those reported from other species including pigs [6], dogs [9], rats [9], and humans [8] though we only showed the patterns of cattle, pigs, dogs, and rats in Table 4. Although cattle and pigs are artiodactyl and phylogenetically more close between them than to other species, the pattern similarity was not much different from comparisons with non-artiodactyls such as dogs, rats, and humans (data not shown), suggesting that these motifs are important for the general function of OR molecules.

**Table 4:** Representative amino acid patterns of the conserved transmembrane motifs of cattle, pig, dog, and rat OR genes

**Pattern No.**	**Transmembrane domain**	**Pattern**
Cattle		
1	TMII	L-x(2,3)-P-M-Y-x-[FL]-[IL]-x(2)-[FL]-[AGS]-x(2)-[DE]
2	TMIII	L-x(1,3)-M-x(2,3)-D-R-x(2)-A-[IV]-x(2)-P-L-x-[HY]-x(3)-[FILMV]
3	TMIII	L-x(2,3)-M-[AGS]-x-D-R-x(2)-A-[IMV]-x(2)-P-[FL]-x-Y
4	TMVI	K-x(3,4)-T-x(2)-[AST]-H-[FILMV]-x(2)-[FILMV]
5	TMVII	P-x-[FILMV]-N-P-x(2)-Y-[ACGST]
Pig		
1	TMII	H-X-P-M-Y-F-F-L-X-[NS]-L-S-[FL]-[AV]-D
2	TMIII	L-X(2,3)-M-[AV]-Y-D-[RS]-F-[LV]-A-I_C-H-P-L-H-Y
3	TMIII	L-X(2,4)-M-[AGS]-X-D-X(2,3)-A-[IV]-X(2)-[LP]-[FIL]
4	TMVI	K-A-[FL]-S-T-C-X-S-H-L-X-V
5	TMVII	P-M-[LM]-N-P-F-[IV]-Y-[NS]-L-X-N-[KR]-[DN]
Dog		
1	TMII	P-M-Y-X-[FL]-L-X(2)-[FL]-[AMS]-X(2)-[DE]
2	TMIII	L-X(3)-M-X(0,1)-Y-X-[FLR]-[LY]-X(2)-[FILV]-[ACS]
3	TMIII	L-X(1,3)-M-X-[FILY]-D-R-X(2)-A-[IV]-[CS]-X-P-L-X-[HY]-X(3)-[ILM]
4	TMVI	K-X-[FL]-[AGHNST]-T-C-X-[AS]-H-X(3)-[AIV]
5	TMVII	N-P-[FILMV]-[IV]-Y-[AGST]-[AILMV]-[KR]-X(2)-[DEKQ]
Rat		
1	TMII	L-[HKNQR]-X-P-M-[FY]-X-[FIL]-L-X(2)-L-X(3)-[DEY]
2	TMIII	M-[AS]-[FLY]-D-R-[FHY]-[AILMV]-A-[IV]-X(2)-P-L-X-[HY]-X(3)-[FILMV]-[DGHKNPRST]
3	TMV	S-Y-X(2)-I-[FILV]-X-[AST]-[FIV]
4	TMVI	K-X-[FILMV]-X-T-C-X-[ACPST]-H-[FILMV]-X(2)-[FILMV]
5	TMVII	P-X-[LMV]-N-P-[FILMV]-X-Y-[ACGST]-X-[KNR]-X-[KNQRT]-[DEKPQ]-[FILMV]

Note: The pattern for dogs and rats was taken from Quignon et al. [9] and pigs from Nguyen et al. [6]. [XYZ] means X or Y or Z. The lower case letter “X” is used as a pattern element to denote any amino acid. X(m) is equivalent to the repetition of X exactly m times. X(m,n) is equivalent to the repetition of X exactly k times for any integer k satisfying: m ≤ k ≤ n.

### Potential odorant specificity of OR subfamilies in cattle

To predict potential target specificity of cattle OR subfamilies in odor perception, we compared the amino acid sequences of the 881 translated cattle OR genes to 2 human ORs [26,27] and 20 mouse ORs [20,21,28-32] with previously described information on odorant specificity. From the analysis, we found that 17 cattle ORs matched ORs of humans and mice with known specificity with at least 60% sequence identity, suggesting that these ORs may share similar olfactory specificities (Table 5). Our analysis also showed that no cattle OR has sequence similarity to OR3A1 and Olfr73; these ORs are known to perceive helional as well as eugenol, which have sweet, hay-like and spicy smells, respectively. It is interesting that *Sus scrofa* also lacks OR3A1, which may be because of the close evolutionary relationship between pigs and cattle. However, Olfr73 was found in pigs. In addition, 3 mouse ORs, Olfr56, Olfr545, and Olfr586, showed relatively lower sequence identity (< 60%) to cattle ORs, which is similar to the analysis result of the pig OR system [6].

**Table 5:** Potential associations between cattle OR gene clusters and odorant recognition

**Cattle OR Locus**	Mouse and human ORs with known odorant recognition*	**Cattle ORs with sequence similarity**	**Amino acid Sequence identity(%)**	**Odorant(s) recognized**	**Perceived odor**
15-47	Olfr2	bOR6F1	89	*n*-aliphatic aldehydes	Fatty
15-48	Olfr653	bOR52F1	83	*n*-aliphatic acids/alcohols	As above
10-27	Olfr749	bOR11B3	82	*n*-aliphatic acids	Rancid, sour, sweaty, fatty
29-28	Olfr151	bOR8H3	81	Acetophenone	Floral/woody
15-45	Olfr480	bOR5F5	80	*n*-aliphatic alcohols	Herbal, woody, orange, rose
3-10	Olfr16	bOR10O3	79	Lyral	Lemony, green
10-22	Olfr49	bOR6N2	79	(−) citronellal	Lemon
15-49	Olfr642	bOR51B3	76	*n*-aliphatic acids	As above
19-24	OR1D2	bOR1S1P	75	Bourgeonal	Lily of the valley
15-47	Olfr154	bOR2A2	74	2-Heptanone	Fruity
15-47	Olfr690	bOR52D1	73	*n*-aliphatic acids/alcohols	As above
26-0	Olfr74	bOR5M3	72	Ethyl vanillin	Vanilla
15-48	Olfr661	bOR53A3P	71	*n*-aliphatic acids/alcohols	As above
15-50	Olfr69	bOR52O9	67	*n*-aliphatic acids/alcohols	As above
11-94	Olfr50	bOR1B10	66	I-carvone	Spearmint, caraway
15-48	Olfr672	bOR52A1	63	*n*-aliphatic acids	Rancid, sour, sweaty, fatty
15-48	Olfr683	bOR53C1	62	*n*-aliphatic acids/alcohols	As above
19-25	Olfr56	bOR1C6	58	Limonene	Lemon
15-49	Olfr586	bOR51C2	51	*n*-aliphatic acids	As above
15-51	Olfr545	bOR52K1	38	*n*-aliphatic dicarboxylic acids	
-	OR3A1	-	-	Helional	Sweet, hay-like
-	Olfr73	-	-	Eugenol	Spicy

* The information on 22 mouse and human ORs with known odorant recognition was from [20,21,26-32]. Dash (−) indicates no match.

## Discussion

Olfaction is essential for mammals to avoid dangers and search for food. Several studies characterizing the OR subgenomes of vertebrates [6-9,33-36] showed significant variations in the number of OR genes among vertebrates, indicating that olfaction machinery in animals was strongly influenced by natural selection [37]. Studying the differences in the genetic makeup of olfaction could provide a window to look into animal evolution associated with environmental changes. In addition, olfaction could be very important in livestock production although it has been poorly understood due to a lack of knowledge regarding the system. Previously, we characterized the OR subgenomes of pigs and reported that the OR gene repertoire in pigs was highly expanded [6]. In this subsequent study, we carried out detail analyses of the OR subgenome of cattle, one of the most important livestock species and another artiodactyl.

Niimura and Nei previously reported the identification of 2,129 OR related sequences (970 functional, 182 truncated and 977 pseudo genes) for the cattle genome using the genome assembly, bosTau2 [10]. However, the accuracy of the results seems to be affected by the quality of the assembly and the analysis were mainly limited to the gains and losses of OR genes. Therefore, we reanalyzed the OR repertoire of cattle using the current genome assembly of *B. taurus* using conserved OR motifs and 24,809 OR protein sequences available from NCBI. As a result, we identified and characterized 1,071 OR-related sequences and their genomic distributions.

### General characteristics of artiodactyl OR system from cattle and pigs

When we compared the structural characteristics of OR gene clusters among cattle, pigs, humans, mice, rats, and dogs, we did not observe any distinctive trends or patterns that reflected the size of the OR gene repertoire (Additional file 10). However, the number of OR genes per cluster was related to the size of the OR gene repertoire, indicating that an increase in OR gene numbers in cattle during evolution was not due to an increase in the number of OR clusters, but was more likely due to an increase in gene numbers within clusters. Moreover, the number of nonfunctional OR clusters consisting of only OR pseudogenes without functional genes was high in the cattle genome with 11 clusters, while only 1 cluster was identified in pigs [6]. This suggests that there is significant variation in the genetic component of OR systems among artiodactyl species, indicating that the selection pressure for maintaining the integrity of OR genes was lower in cattle comparing to pigs.

### Evolutionary relationships of OR systems among mammals

**Figure 3:**
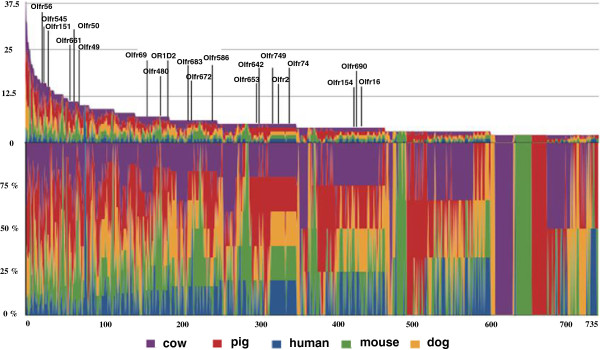
**Comparison of OR gene similarity among humans, dogs, mice, pigs, and cattle by clustering analysis of OR genes on the basis of amino acid sequence similarity.** The Y-axis of the upper graph shows the number of OR genes in each cluster ranging from 2 to 43 genes. The X-axis of the lower graph indicates the cluster number, with 751 clusters. The Y-axis of the lower graph indicates the percentage of OR genes of each species within the cluster. The OR genes of different species are indicated by different colors.

To understand the evolutionary relationships between OR genes of cattle, pigs, humans, mice, and dogs, we combined 4,582 OR gene sequences from these 5 species and performed clustering according to their protein sequence similarity (Figure 3). Using a cutoff of more than 60% sequence identity to group sequences together into a single cluster, 751 clusters were generated according to OR gene sequence similarity among cattle, pigs, humans, mice, and dogs. OR genes of different species in the same cluster may recognize similar odorant substances because it has been reported that ORs sharing more than 60% in their sequence homology bind to odorants with similar chemical structures [20,21].

We observed that 26% of the OR clusters (n = 199) contained genes which were common to 4 species, and these were the most common OR genes in respect to OR sharing among species (Additional file 11). The second most common type of cluster were those shared by 3 species, consisting of 23% of the OR clusters (n = 170). We found 73.6% (n = 53) of the 72 cattle-specific OR genes were functional genes, indicating that cattle contains more unique OR genes than humans and dogs (Table 6). The number of clusters specific to cattle, pigs, humans, mice, and dogs was 30, 35, 3, 36, and 11, respectively (Additional file 11). The presence of unique or common OR genes across different species reflects diversification or maintenance of orthologous genes from common ancestors during evolution of the species. Consistent with this, we found that the protein sequences of 13 functional OR genes in cattle were highly similar (>70%) to those of OR pseudogenes of other species (Additional file 12).

**Table 6:** Number of common or unique OR genes among cattle, pig, human, mouse, and dog OR repertoires

**Species sharing the same OR gene clusters**	**Number of OR genes belonging to the species common clusters**				
	**Cattle**	**Pig**	**Human**	**Mouse**	**Dog**
Cattle, pig, human, mouse, dog	284	313	166	250	217
Cattle, pig, mouse, dog	178	217	-	161	167
Cattle, pig, human, dog	59	79	52	-	55
Cattle, pig, human, mouse	48	62	28	32	-
Cattle, human, mouse, dog	24	-	24	27	25
Pig, human, mouse, dog	-	38	27	32	37
Cattle, pig, dog	74	103	-	-	68
Cattle, pig, mouse	41	67	-	66	-
Cattle, mouse, dog	27	-	-	25	27
Pig, mouse, dog	-	21	-	23	18
Cattle, dog	17	-	-	-	20
Pig, dog	-	15	-	-	15
Cattle, pig	63	147	-	-	-
Cattle	72	-	-	-	-
Pig	-	98	-	-	-
Human	-	-	22	-	-
Mouse	-	-	-	116	-
Dog	-	-	-	-	27

Note: Sequences with more than 60% of amino acid sequence identity were clustered together.

The number of cattle OR genes common to only both cattle and pigs (n = 63) was much larger than those common to only both cattle and dogs (n = 17) (Table 6). This could be due to the closer phylogenetic relationship of cattle to pigs than to dogs. However, this also could be due to the higher similarity in environmental factors for their survival between cattle and pigs than cattle and dogs. For example, cow’s grazing and pig’s rooting for foods probably share more similarity than the food searching behavior of dogs.

### Copy number variations of OR genes

Jessica et al. reported a homozygous deletion of 6 olfactory receptor genes in a subset of individuals with beta-thalassemia which was caused by a 118 kb deletion involving β-globin and the neighboring olfactory receptor genes [38]. It would be interesting to evaluate individual CNVs of OR genes due to deletions or duplications in cattle in a large scale although it will be difficult to accurately illuminate them without proper resources such as high-density chromosome arrays. However, it is interesting that 40% of the tested OR loci in this study showed CNVs. This indicates that the copy number variations of OR genes in cattle are very common. The diversity of OR genes in cattle could be very high and lead to individual or breed specific differences in olfaction capacity.

## Conclusions

We report here a genome level analysis of OR genes in cattle using conserved motif sequences specific to OR genes. Our results can be utilized as comparative information to understand the genetic organization of OR genes in mammals and contribute to understanding of the characteristics of chemosensory responses in cattle.

## Competing interests

The authors declare that they have no competing interests.

## Authors’ contributions

KL and DTN carried out the bioinformatics analyses and classification of bovine OR genes, interpreted the data, and drafted the manuscript. MC evaluated the results of the bioinformatics analyses. SYC, JHK, HD, HGS, KS, and TC provided helpful ideas and critical discussion for the analysis. CP was involved in project planning, discussion, and writing of the manuscript as a project principal investigator. All authors read and approved the final manuscript.

## Supplementary Material

Additional file 1 **Primer pairs used to test copy number variation of 10 bovine OR genes.** Table describing the primer information to amplify 10 selected bovine OR genes using locus-specific PCR from 22 individuals of three different breeds. Click here for file

Additional file 2 **Criteria for pattern recognition of cattle OR genes by using the PRATT program.** Table describing parameters and values for pattern recognition of cattle OR genes. Click here for file

Additional file 3 **Analysis of the number of functional OR genes and subfamily distribution per cluster.** Table describing the relationship between number of OR gene clusters with number of functional OR genes as well as number of subfamilies with number of clusters. Click here for file

Additional file 4 **Cattle OR gene coordinates in the cattle genome assembly UMD 3.1.** Table listing positions of functional and pseudo OR gene sequences in the cattle genome. Click here for file

Additional file 5 **Comparison of family and subfamily diversity of OR genes among cattle, pigs, humans, dogs, mice, and rats.** Table showing the results of comparative analysis of the number of classes, families, and subfamilies among 6 species including cattle, pigs, humans, dogs, mice, and rats. Click here for file

Additional file 6 **The number of OR gene subfamilies according to their OR gene numbers.** Table showing the number of OR gene subfamilies according to their OR gene numbers (1 to 63) within the subfamilies. Click here for file

Additional file 7 **OR gene subfamilies with gene number expansion with more than 20 genes.** Table showing the sharing of expanded cattle OR gene subfamilies with expanded OR gene families across species. Click here for file

Additional file 8 **Distribution of OR gene duplications in the cattle genome.** Table showing the distribution of OR genes duplicated in the cattle genome. Click here for file

Additional file 9 **Confirmation of OR gene duplications in cattle genome by PCR amplification.** Figure showing PCR amplifications of two duplicated OR genes (bOR7A17A and bOR7A17B) obtained from genomic DNAs of Korean native cattle and Black Angus. Lane M, size marker; 1, bOR7A17A (Hanwoo); 2, bOR7A17A (Black Angus); 3, bOR7A17B (Hanwoo); 4, bOR7A17B (Black Angus); 5, Negative control. Click here for file

Additional file 10 **Comparison of the structural characteristics of OR genes among cattle, pigs, humans, mice, rats and dogs.** Table listing number of clusters, number of genes per cluster, and number of clusters with only pseudogenes for cattle, pigs, humans, dogs, mice, and rats. Click here for file

Additional file 11 **Number of OR genes within each cluster (n = 751) in Figure** 3**.** Number of OR genes within each cluster (n = 751) from the comparison of OR gene similarity among humans, dogs, mice, pigs and cattle using cluster analysis in Figure 3. Click here for file

Additional file 12 **The amino acid sequence similarity between functional OR genes of cattle and the pseudogenes of other species.** Table listing 13 pairs of cattle functional OR genes and pseudogenes of other species with high protein sequence homology (>70%). Click here for file
