# Supplementary material for: Analysis of cattle olfactory subgenome: the first detail study on the characteristics of the complete olfactory receptor repertoire of a ruminant
Source: BMC Genomics. 2013 Sep 2;14:596. doi: 10.1186/1471-2164-14-596 (PMC3766653; doi:10.1186/1471-2164-14-596)
Supplement: Additional file 1 — Primer pairs used to test copy number variation of 10 bovine OR genes. Table describing the primer information to amplify 10 selected bovine OR genes using locus-specific PCR from 22 individuals of three different breeds. [file 1471-2164-14-596-S1.docx]

**Additional file 1.** Primer pairs used to test copy number variation of 10 bovine OR genes.

| **Primer names for PCR** | **Sequence (5’- 3’)** | **Ta (^o^C)** | **Size (bp)** |
| --- | --- | --- | --- |
| bOR1O1A^a^ | F-GCTAGTGTAAGATCTCTCATTACC | 60 | 1133 |
|  | R-GACAGGAAGTTCACCAGCATC |  |  |
| bOR1O1B^a^ | F-TCCTCCTCTTGGCAGAGAATG | 60 | 1240 |
|  | R-GACAGGAAGTTCACCAGCATC |  |  |
| bOR1O2^b^ | F-CTTGGGCTATGGAATTGGAACA | 55 | 1163 |
|  | R-AAGTATCTTGATTAAGCAGAGG |  |  |
| bOR1O4^b^ | F-CTTGGGCTATGGAATTGGAACC | 55 | 1163 |
|  | R-AAGTATCTTGATTAAGCAGAGA |  |  |
| bOR2AK2^c^ | F-CCACAGGAACAAAATAGGAATG | 55 | 1169 |
|  | R-TTCTGGAAAGGAATATGAGAAG |  |  |
| bOR2AK3^c^ | F-TGCCACAGGAACAAAATAAGAA | 54 | 1171 |
|  | R-CTTTCTGGAAAAAGAATACGAG |  |  |
| bOR7A17A^d^ | F- CTTCTCTGGTGGCTCAGTAGTA | 55 | 826 |
|  | R- AAGGACAGGTTGGAGAGGAAGA |  |  |
| bOR7A17B^d^ | F- CTTGGTGATGTACACTGTGGTC | 61 | 414 |
|  | R- CACACAGATAGCGGTGAATGTC |  |  |
| bOR9M7^e^ | F-ATATTGGCATGAATTAGATAC | 56 | 541 |
|  | R-TTTCTGCTCATGATGGTTGTA |  |  |
| bOR9M8^e^ | F-ATATTGGCATGAATTAGATAG | 55 | 551 |
|  | R-TTCCTGCTCATGATGGTTGTG |  |  |

Note: The same superscript indicates pairs of ORs with nucleotide sequence identity greater than 99%. F, Forward primers; R, Reverse primers; Ta, primer annealing temperature.
