# Supplementary material for: Analysis of cattle olfactory subgenome: the first detail study on the characteristics of the complete olfactory receptor repertoire of a ruminant
Source: BMC Genomics. 2013 Sep 2;14:596. doi: 10.1186/1471-2164-14-596 (PMC3766653; doi:10.1186/1471-2164-14-596)
Supplement: Additional file 3 — Analysis of the number of functional OR genes and subfamily distribution per cluster. Table describing the relationship between number of OR gene clusters with number of functional OR genes as well as number of subfamilies with number of clusters. [file 1471-2164-14-596-S3.docx]

**Additional file 3.** Analysis of the number of functional OR genes and subfamily distribution per cluster.

| Functional OR genes  per cluster^1^ | | |  | Clusters  per subfamily^2^ | | |  | | Subfamilies  per cluster^3^ | |
| --- | --- | --- | --- | --- | --- | --- | --- | --- | --- | --- |
| No. of OR genes | No. of clusters |  | | | No. of clusters | No. of subfamilies | |  | No. of subfamilies | No. of clusters |
| 0 | 5 |  | | | 1 | 228 | |  | 1 | 16 |
| 1 | 7 |  | | | 2 | 31 | |  | 2 | 5 |
| 2 | 6 |  | | | 3 | 6 | |  | 3 | 6 |
| 3 | 7 |  | | | 4 | 3 | |  | 4 | 3 |
| 4 | 1 |  | | | 5 | 2 | |  | 5 | 2 |
| 5 | 2 |  | | | 6 | 1 | |  | 6 | 3 |
| 6 | 1 |  | | | 7 | 1 | |  | 7 | 1 |
| 9 | 1 |  | | |  |  | |  | 8 | 2 |
| 11 | 1 |  | | |  |  | |  | 9 | 2 |
| 12 | 2 |  | | |  |  | |  | 10 | 1 |
| 14 | 1 |  | | |  |  | |  | 12 | 2 |
| 16 | 1 |  | | |  |  | |  | 13 | 1 |
| 18 | 2 |  | | |  |  | |  | 19 | 1 |
| 20 | 1 |  | | |  |  | |  | 27 | 1 |
| 23 | 1 |  | | |  |  | |  | 29 | 1 |
| 26 | 1 |  | | |  |  | |  | 45 | 1 |
| 38 | 1 |  | | |  |  | |  | 51 | 1 |
| 56 | 1 |  | | |  |  | |  |  |  |
| 57 | 1 |  | | |  |  | |  |  |  |
| 60 | 2 |  | | |  |  | |  |  |  |
| 66 | 1 |  | | |  |  | |  |  |  |
| 67 | 1 |  | | |  |  | |  |  |  |
| 116 | 1 |  | | |  |  | |  |  |  |
| 122 | 1 |  | | |  |  | |  |  |  |

^1^Number of OR gene clusters with 0 to 122 functional OR genes.

^2^Number of subfamilies whose members are encoded in 1 to 7 clusters.

^3^Number of clusters that encode members of 1 to 51 subfamilies.
