# Supplementary figures and images for: Analysis of cattle olfactory subgenome: the first detail study on the characteristics of the complete olfactory receptor repertoire of a ruminant
Source: BMC Genomics. 2013 Sep 2;14:596. doi: 10.1186/1471-2164-14-596 (PMC3766653; doi:10.1186/1471-2164-14-596)

## Slide 1
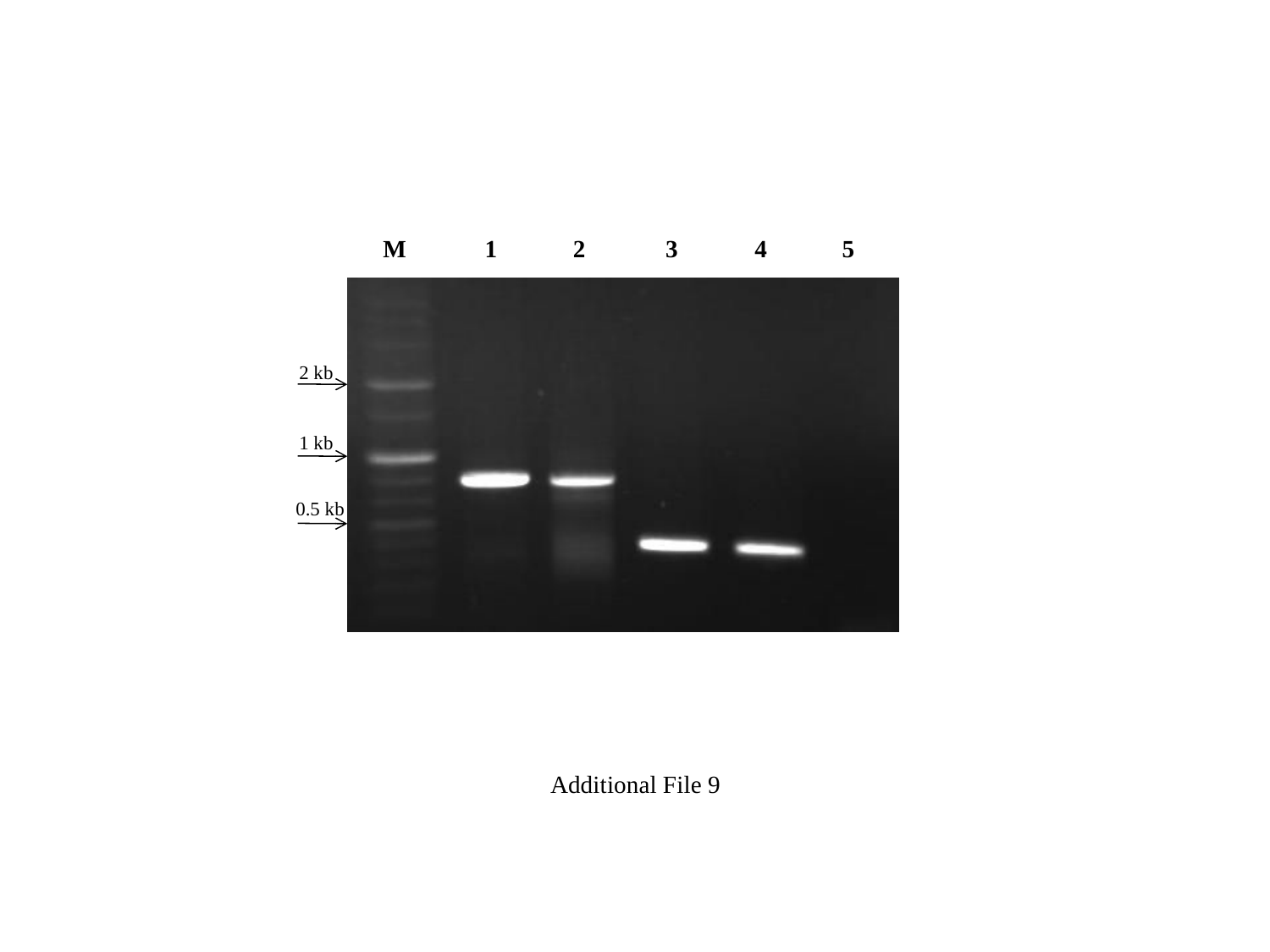

M
1
2
3
4
5
2 kb
1 kb
0.5 kb
Additional File 9

Supplement: Additional file 9 — Confirmation of OR gene duplications in cattle genome by PCR amplification. Figure showing PCR amplifications of two duplicated OR genes (bOR7A17A and bOR7A17B) obtained from genomic DNAs of Korean native cattle and Black Angus. Lane M, size marker; 1, bOR7A17A (Hanwoo); 2, bOR7A17A (Black Angus); 3, bOR7A17B (Hanwoo); 4, bOR7A17B (Black Angus); 5, Negative control. [file 1471-2164-14-596-S9.ppt]
